# Phytochemical Content and Antidiabetic Properties of Most Commonly Used Antidiabetic Medicinal Plants of Kenya

**DOI:** 10.3390/molecules28207202

**Published:** 2023-10-20

**Authors:** Felix Wambua Muema, Consolata Nanjala, Millicent Akinyi Oulo, Phurpa Wangchuk

**Affiliations:** 1 CAS Key Laboratory of Plant Germplasm Enhancement and Specialty Agriculture, Wuhan Botanical Garden, Chinese Academy of Sciences, Wuhan 430074, China; fwambua83@gmail.com (F.W.M.); millicentoulo@gmail.com (M.A.O.); 2 University of Chinese Academy of Sciences, Beijing 100049, China; 3 College of Science and Engineering, James Cook University, Cairns, QLD 4870, Australia; consolata.nanjala@my.jcu.edu.au; 4 Australian Tropical Herbarium, James Cook University, Cairns, QLD 4878, Australia; 5 Centre for Molecular Therapeutics, Australian Institute of Tropical Health and Medicine, James Cook University, Building E4, McGregor Rd., Smithfield, Cairns, QLD 4878, Australia

**Keywords:** diabetes, phytoconstituents, *Allium cepa*, antidiabetic, α-glucosidase, ethnomedicine, traditional medicine

## Abstract

Traditional medicinal plants have been used for decades in folk medicines in the treatment and management of several ailments and diseases including diabetes, pain, ulcers, cancers, and wounds, among others. This study focused on the phytochemical and antidiabetic activity of the commonly used antidiabetic medicinal species in Kenya. Phytochemical profiling of these species revealed flavonoids and terpenoids as the major chemical classes reported which have been linked with strong biological activities against the aforementioned diseases, among others. However, out of the selected twenty-two species, many of the natural product isolation studies have focused on only a few species, as highlighted in the study. All of the examined crude extracts from thirteen antidiabetic species demonstrated strong antidiabetic activities by inhibiting α-glucosidase and α-amylase among other mechanisms, while nine are yet to be evaluated for their antidiabetic activities. Isolated compounds S-Methylcysteine sulfoxide, quercetin, alliuocide G, 2-(3,4-Dihydroxybenzoyl)-2,4,6-trihydroxy-3 (2*H*)-benzofuranone, Luteolin-7-*O*-D-glucopyranoside, quercetin, 1,3,11*α*-Trihydroxy-9-(3,5,7-trihydroxy-4*H*-1-benzopyran-7-on-2-yl)-5*α*-(3,4-dihydroxy-phenyl)-5,6,11-hexahydro-5,6,11-trioxanaphthacene-12-one and [1,3,11*α*-Trihydroxy-9-(3,5,7-trihydroxy-4*H*-1-benzopyran-7-on-2-yl)-5*α*-(3,4-dihydroxy-phenyl)-5,6,11-hexahydro-5,6,11-trioxanaphthacene-12-one]-4′-*O*-D-gluco-pyranoside from *Allium cepa* have been found to exhibit significant antidiabetic activities. With the huge number of adults living with diabetes in Kenya and the available treatment methods being expensive yet not so effective, this study highlights alternative remedies by documenting the commonly used antidiabetic medicinal plants. Further, the study supports the antidiabetic use of these plants with the existing pharmacological profiles and highlights research study gaps. Therefore, it is urgent to conduct natural products isolation work on the selected antidiabetic species commonly used in Kenya and evaluate their antidiabetic activities, both in vitro and in vivo, to validate their antidiabetic use and come up with new antidiabetic drugs.

## 1. Introduction

Numerous natural substances found in plants have significant bioactive characteristics. These substances may offer substitutes for existing drugs and open up a wide window for the development of novel drugs. Humankind has been attempting to control diseases in various ways from time immemorial, and numerous medicinal plants have made significant contributions in this respect from time to time. Plant-derived remedies have been used in traditional medicine for thousands of years in most parts of the world, and are the earliest known therapies to mankind [1]. The world’s plant biodiversity is the most abundant source of herbal medicine, and around 80% of the world’s population relies on plant-based medicines, which have been utilized as traditional healthcare systems since ancient times. The bioactive chemicals in these herbal plants have several physiological impacts on the human body and can be used to cure a variety of ailments. Many of today’s contemporary pharmaceuticals are based on natural chemicals originating from plants [2].

Diabetes mellitus (DM) is a chronic condition that occurs when the body no longer generates enough insulin or when the insulin produced cannot be used adequately [3]. Diabetes is classified into two types: type 1 diabetes (T1DM), which is an autoimmune illness characterized by insulin insufficiency, and type 2 diabetes (T2DM), which is characterized by inadequate insulin action [4]. T1DM (5–10% of all diabetes) is caused by the cellular-mediated autoimmune destruction of insulin-producing pancreatic β-cells, resulting in insulin insufficiency. Furthermore, T2DM (90–95% of all diabetes) is caused by a continual reduction in insulin secretion, which is generally accompanied by insulin resistance [5,6]. Multiple factors, including genetic predisposition, lifestyle, and gut microbial dysbiosis, have been implicated in the pathophysiology of diabetes. Polydipsia, polyphagia, polyuria, excessive weariness, lack of energy, and weight loss are all signs of type 1 diabetes. Type 2 diabetes has its own set of symptoms, including increased thirst and hunger, exhaustion, frequent urination, unintentional weight loss, darker skin, and frequent infections, most commonly in the armpits and neck [7]. Prolonged hyperglycemia exposure can cause irreparable damage to various organs, including the eyes, nerves, and kidneys. Meanwhile, it can cause heart disease, nephropathy, stroke, retinopathy, osteoporosis, and other complications, resulting in morbidity and mortality [8]. Over the years, diabetes cases have risen at an alarming rate, hence an immediate response to counter the increasing number of cases is required. Globally in 2015, there were 425 million diabetic adults and the number is predicted to rise to 642 million by 2045 [9].

Traditional medicine used to be the predominant medical system available to millions of people in Africa in both rural and urban regions before the advent of cosmopolitan medicine. There are strong indications that traditional healthcare systems are still in use by a majority of people not only in Africa, but across the world [10]. While the rest of the world is rapidly modernizing traditional medicines by evaluating their phytochemical and pharmacological profiles to ascertain their uses, Africa, specifically Kenya, lags behind. By rigorously evaluating the existing literature on the phytochemistry, antidiabetic properties, potential mechanisms of action, and toxicology of select antidiabetic medicinal plants, this review seeks to build a connection between ethnobotanical applications and scientific studies. Additionally, to properly exploit the select species, these analyses also identify numerous gaps in the research and establish a baseline for future research projects in the development of antidiabetic drugs.

## 2. Diabetes Mellitus Mechanism of Action

Antidiabetic drugs are categorized into α-amylase and α-glucosidase inhibitors, glucagon-like peptide 1 receptor agonists, meglitinides, sulfonylureas, dopamine-2 agonists, bile acid sequestrants, sulfonylureas, and insulin and its analogs among other classes based on their targets [11,12,13]. Natural products, such as traditional herbal formulas, plant extracts, and their chemical ingredients, have recently sparked great interest in diabetes treatment. Natural products compounds, similarly to known pharmaceuticals, display their therapeutic impact by interacting with diabetes-related macromolecule targets [14]. Some phytoconstituents have been reported to inhibit several diabetes pathogenesis pathways. For instance, flavonoids are reported to inhibit cyclo-oxygenase (COX-1/2) [15], while alkaloids inhibit adenosine 5′-monophosphate-activated protein kinase (AMPK) [16,17]. AMP: ATP and ADP: ATP ratios both affect cellular energy levels and ATP affects both cellular growth and survival. Previous studies show that activation of AMPK increases ATP-generation rate while the rate of ATP-consumption decreases. Therefore, through either a catabolic pathway for producing ATP or an anabolic pathway for consuming it, AMPK can restore energy balance [18].

Further, polyphenols have demonstrated their inhibition of DPP-4 [16]. Some phytochemicals, including flavonoids, alkaloids, bromophenols, and diterpenes among others have been discovered as PTP1B inhibitors [19]. PTP1B is a member of the protein-tyrosine phosphatases (PTPs) family, which is considered a viable therapeutic target for the treatment of type 2 diabetes. It is a negative regulator of the insulin and leptin signaling pathways and is found to be a promising prospective therapeutic target, particularly for type 2 diabetes treatment [20,21]. Acarbose, α-amylase and α-glucosidase inhibitors are used to slow down the rate of carbohydrate metabolism and control blood sugar levels in diabetic patients [22].

The peroxisome proliferator-activated receptor (PPAR) has three isotypes, α, δ, and γ, and is a nuclear receptor superfamily that may have an impact on insulin sensitivity, inflammation, lipid metabolism, and insulin secretion in the treatment of diabetes [23]. The gamma isotype, PPARγ could be involved in lipid mobilization, glucose metabolism, the inflammatory response, and the generation and release of adipokines [24,25]. Investigations have revealed that PPARγ ligands may boost triglyceride storage in fat, which has been linked with insulin resistance, as well as modulation of adipocyte-secreted hormones, hence they are a target for diabetes mitigation [24]. Insulin receptor α-subunits receive insulin signals which activate the tyrosine kinase of β-subunits to induce intracellular auto-phosphorylation at Tyr1158, Tyr1162, and Tyr1163. Upon activation, the insulin receptors cause PI3K to phosphorylate PIP2, and PIP3 in turn activates PDK1/2. Once AKT is phosphorylated as a result of the signal, the downstream AS160 causes GLUT4 translocation and glucose uptake into the cells [26,27]. Phytochemicals, such as rutin isolated from *Toona sinensis* Roem, may increase IRK activation to facilitate skeletal tissue glucose absorption and, as a result, improve insulin resistance by lowering blood glucose levels in diabetes patients [26]. Glycogen kinase, which is found in liver cells, primarily regulates glycogen storage in the liver. Insulin and glucagon can activate the transport of glycogen kinase by glucose transporters, modifying the amount of glycogen kinase in the cytoplasm of hepatocytes and managing the intracellular glucose content for the management of type 2 diabetes. Therefore, glycogen kinase agonists are a new class of antidiabetic drugs [28].

This review, therefore, focuses on the ethnobotanical uses, phytochemistry (isolated chemical constituents), and antidiabetic activity of the most commonly used plants to treat diabetes in Kenya. Data on the commonly used plant species were obtained by searching scientific online databases including PubMed, Google Scholar, Web of Science, Science direct, Elsevier, and SciFinder. We also utilized websites such as www.sciencedirect.com (accessed on 8 August 2023) and www.eflora.org (accessed on 3 August 2023) to explore pertinent information, employing various keywords such as “*A. nilotica* extract as traditional medicine”, “antidiabetic activity of *A. nilotica*”, “phytochemistry of *A. nilotica*”, among others. The chemical structures of the antidiabetic compounds were depicted using ChemDraw resources. We used a variety of sources for our examination of the literature, including abstracts, full-text original articles, dissertations, and PhD theses. To properly cite all relevant literature, we employed EndNote 20.2.1 software.

## 3. Ethnobotany

Numerous ethnopharmacological research studies have revealed a vast variety of medicinal plant species utilized by Kenyan communities to treat and manage various ailments. Most of these medicinal plants are utilized by the Maasai community from Kenya which is highly recognized and esteemed for their ethnomedicines. The selected antidiabetic medicinal plant species are used traditionally not only to treat and prevent diabetes but also other human diseases. Other ethnobotanical uses of these species are listed in Table 1. The medicinal preparation procedures vary with different communities as well as the dosage dispensation. Different parts of each of the select species can be used to treat different diseases [29]. Further, different plant parts of the same plant species can be used in combinations as well as different plant species [30]. For instance, the paste of the fresh leaves and latex of *Euphorbia tirucalli* are mixed and diluted in water and taken once a day to treat cancer, while the latex is boiled in mustard oil in a ratio of 1:5, and drops of the mixture are used to treat cure ear problems [29,31]. At high doses, *E. tirucalli* is irritant, bitter, and emetic. *Allium cepa* and *Allium sativum* are used in combination to treat and manage diabetes [32]. The leaves of *A. wilkesiana* are boiled in water and the decoction is used to treat diabetes mellitus in adults. It is also used to treat other diseases such as fungal infections in newborns and hypertension [33]. *A. wilkesiana* is also used traditionally to treat fungal and bacterial infections, gastrointestinal disorders, and neonatal jaundice [34].

Manihot esculenta, commonly known as cassava in Kenya, is used as a source of both nutrition and medicine. The roots are consumed as a source of nutrients, rich in starch, while a paste prepared from the leaves is used for medicinal purposes, including to treat diabetes, headaches, and hypertension [35,36]. Notably, roots are the most-used plant parts in the preparation of decoctions, followed by leaves. This however raises concerns for the medicinal plants’ conservation. According to most communities in Kenya, there are regulations of ethnomedicinal plant uses to ensure that medicinal plants are not overused or misused. In most communities, any medicinal plant should not be harvested for more than a third of its roots.

**Table 1:** Ethnobotanical uses of the selected medicinal species.

Species and Family	Ethnomedicinal Uses
*Acacia nilotica* (L.) Willd. ex Delile (Mimosoideae)	The trunk bark is used to treat diarrhea, colds, bleeding piles, bronchitis and leucoderma [37]. The roots are used to treat cancers and tumors, diabetes, and tuberculosis [38]. Pods are used as antihypertensive and antispasmodic, anti-fertility, and astringent [38].
*Acalypha wilkesiana* Müll.Arg. (Euphorbiaceae)	The leaves are used to treat diabetes mellitus, malaria, hypertension, skin infections, and gastrointestinal disorders [33,34].
*Allium cepa* L. (Amaryllidaceae)	The whole plant is used in healing wounds, treating diabetes, pneumonia, headaches, fever, cough, flu, sore throat, high blood pressure, and rheumatism [39].
*Aloe secundiflora* Engl. (Asphodelaceae)	An infusion from the leaves is used to treat bacterial diseases, ectoparasites, diabetes, fowl typhoid, nose bleeding, malaria, and wounds [40,41].
*Carissa edulis* (Forssk.) Vahl (Apocynaceae)	A decoction prepared from the leaves is used for indigestion, malaria, and abdominal pain in pregnant women. The root is used to treat chest pains, gonorrhea, swollen glands, back pains, diabetes, syphilis, toothache, epilepsy, and sickle cell anemia [42,43].
*Dovyalis abyssinica* (A.Rich.) Warb. (Salicaceae)	The leaves and roots are used to treat and manage ulcers, wounds, throat inflammation, pneumonia, malaria, diabetes, and indigestion [41,44].
*Dracaena steudneri* Schweinf. Ex. Engl. (Asparagaceae)	The leaves are used to treat hernias, asthma, splenomegaly, chest problems, and liver diseases. The stem bark is used to treat liver diseases and measles, and to reduce pain during childbirth [45].
*Euphorbia hirta* L. (Euphorbiaceae)	A decoction of the whole plant is used to treat respiratory system disorders, diabetes, ulcers, amebic dysentery, gonorrhea, and several types of cancers [46].
*Euphorbia tirucalli* L. (Euphorbiaceae)	The latex treats cancers, toothaches, skin diseases, intestinal parasites, snake bikes, coughs, scorpion stings, asthma and ear problems [29], and syphilis [47]. The leaves are used to treat skin problems, diabetes, diarrhea, nose ulcers, and hemorrhoids. The stems are used for thorn extraction, and treating swelling, leprosy, paralysis, colic, and gastric problems. The roots are used to treat rheumatism [29].
*Faurea saligna* Harv. (Proteaceae)	Used to treat sores and wounds, diabetes, fungal infections, candidiasis, and stomach problems [41].
*Lactuca inermis* Forssk (Asteraceae)	A decoction of the leaves is used to treat joint pain, amebiasis, throat and nose diseases, and diabetes [48].
*Manihot esculenta* Crantz (Euphorbiaceae)	The leaves are used in treating wounds, diabetes, headache, pain, and hypertension [49].
*Myrsine africana* L. (Primulaceae)	Used to treat diarrhea, toothache, rheumatism, diabetes, and pulmonary tuberculosis [48,50,51].
*Persea americana* Mill. (Lauraceae)	Traditionally used to treat rheumatism, bronchitis, urinary infections [52], hypertension, diabetes, stomach aches, and bronchitis [53].
*Prunus africana* (Hook.f.) Kalkman (Rosaceae)	Used to treat diabetes, high blood pressure, stomach problems, chest pains, fever, and malaria [54,55,56].
*Rhamnus prinoides* L’Hér. (Rhamnaceae)	A decoction of the leaves is used to treat pneumonia, common colds, chest pain, tonsils, diabetes, back pain, and malaria [57].
*Rhamnus staddo* A.Rich. (Rhamnaceae)	In East Africa, the stems, roots, fruits and leaves are used to treat malaria, diabetes, and endometritis [41,58].
*Rotheca myricoides* (Hochst.) Steane and Mabb. (Lamiaceae)	A decoction prepared from the leaves is used to treat and manage diabetes, arthritis, rheumatism, gonorrhea, typhoid, malaria, epilepsy, and cancer [59].
*Trimeria grandifolia* (Hochst.) Warb. (Salicaceae)	The roots are used to treat back pain, and a decoction of the stem is used to manage postpartum weakness, malaria, and diabetes [41].
*Urtica massaica* Mildbr (Urticaceae)	A decoction of the leaves is used to treat cancer, diabetes, and malaria [41,60].
*Warburgia ugandensis* Sprague (Canellaceae)	The leaves and stem bark are used to treat pains, coughs, malaria, colds, toothache, constipation, stomachache, and diabetes [61,62,63].
*Zanthoxylum usambarense* (Engl.) (Rutaceae)	Decoctions of the leaves and roots are taken to treat stomachache, colds, toothache, and diabetes [64].

## 4. Phytochemistry

**Figure 1:**
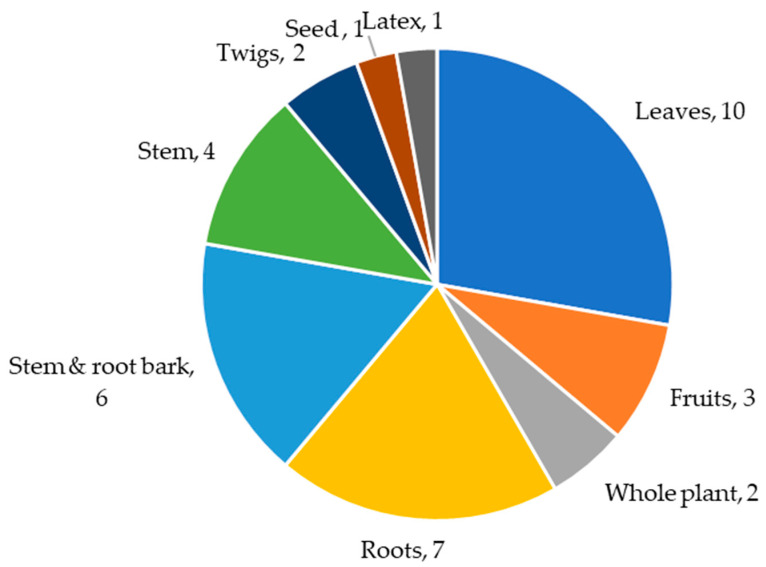
Different plant parts used for the isolation of phytoconstituents.

**Figure 2:**
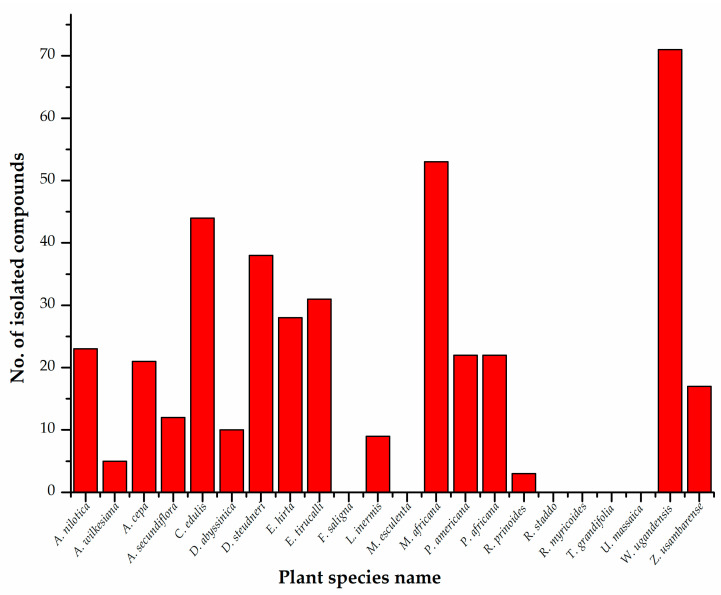
Number of isolated chemical constituents from each species.

Phytochemical studies have significantly contributed to the knowledge of bioactive chemical constituents. The phytochemistry of the selected antidiabetic plant species has revealed that the majority of the species contain various chemical classes. The reported chemical compounds have been isolated from different plant parts. The leaves are the most-used plant part, followed by the bark, as shown in Figure 1. Out of the selected plant species, six have no records of isolated compounds even though the extracts have exhibited biological activities, however, compounds have been detected in the crude extracts. *W. ugandensis* has the highest record of isolated chemical constituents. Figure 2 shows the number of isolated phytoconstituents of each species. This identifies a gap in the isolation of the bioactive constituents from these species. These medicinal plant species include *Faurea saligna*, *Manihot esculenta*, *Rhamnus staddo*, *Rotheca myricoides*, *Trimeria grandifolia* and *Urtica massaica*. Flavonoids, lignans, and terpenoids are the most isolated chemical classes from the selected species.

### 4.1. Terpenoids

Terpenoids are one of the most prevalent and structurally varied classes of natural chemical constituents. Terpenoids are then sub-grouped into monoterpenoids, diterpenoids, triterpenoids, sesquiterpenoids, and polyterpenoids. Terpenoids exhibit numerous significant biological activities, including antidiabetic, anti-inflammatory, hepatoprotective, neuroprotective, and cardioprotective activities [65,66], hence they are targeted for drug development research. Among the selected medicinal plant species, *C. edulis*, *M. africana*, *P. africana*, *R. prinoides*, and *W. ugandensis* have reported terpenoids which have been found to exhibit significant pharmacological activities. Tigliane-type diterpenoids with a transfused 5/7 ring system isolated from *E. tirucalli* were reported to exhibit potent inhibitory activity against p-glycoprotein in HepG2/ADR cells [67]. Manguro et al. isolated three oleanane triterpenoids, taraxerone, taraxerol and myricadiol, from the leaves of *M. africana* collected in Kenya [68]. Sesquiterpenoids have been reported as the dominant chemical class of *W. ugandensis*. Xu et al. isolated nine drimane-type sesquiterpenoids from the stem bark of *W. ugandensis* collected in Uganda [69]. Weng et al. isolated new diterpenoids from aerial parts of *E. tiruvalli* collected in China [67].

### 4.2. Flavonoids

Flavonoids are regarded as dietary supplements that fight disease and promote health. They are recorded in the majority of plant species. They have been discovered to demonstrate a wide range of biological activities, including antiviral, antidiabetic, anticancer, anti-inflammatory, and antibacterial activities, among other pharmacological properties [70]. Flavonoids are also known to be strong antioxidants [71]. Kaempferol and quercetin were reported in most of the selected antidiabetic medicinal species. Lima et al. isolated flavonoids and their glycosides from the roots of *Euphorbia tirucalli* collected in Brazil [72]. Nchiozem-Ngnitedem et al. also isolated bioactive flavonoids and their glycosides from the seeds of *Dracaena steudneri* collected in Kenya [73].

### 4.3. Sterols

Sterols are mostly known for their immune system-modulating and anti-inflammatory activities [74]. However, they have been also reported to exhibit antidiabetic properties. Stigmaterol is a known steroid isolated in many plant species and has been reported in our selected Kenyan antidiabetic medicinal species. Pharmacological studies have shown its antidiabetic potential in targeting GLUT4 glucose transporters by boosting GLUT4 translocation and expression [75].

### 4.4. Lignans

Lignans are polyphenols that are found in vegetables, seeds, legumes, fruits, and grains. Lignans exhibit various biological activities such as treating cancer [76] and preventing diseases such as type 2 diabetes mellitus [77], cardiovascular diseases [78], and coronary heart diseases [79]. Lignans have been reported in most of the studied species. Zhang et al. isolated new lignans from *E. hirta* plant, namely euphorhirtins A–D and 5-methoxyvirgatusin among other previously isolated lignans, as listed in Table 2. Among the isolates, niranthin and 7-hydroxy-hinokinin exhibited inhibitory activities against the cancer cell line Hep G2 [80]. Kaunda et al. isolated three furofuran lignans glycosides from the root barks of *Carissa edulis* collected in Kenya [81].

### 4.5. Alkaloids

Alkaloids are a large group of naturally occurring organic materials that have one or more nitrogen atoms (in some cases amino or amido) in their structures with extraordinarily diverse chemical configurations, including heterocyclic ring systems. Even at very low levels, they exhibit potent biological effects on both animal and human cells [82]. *Zanthoxylum usambarense* has been reported to possess bioactive alkaloids isolated from the roots and stem barks from samples collected in Kenya [64,83]. Rasmussen et al. isolated alkaloids from the leaves and twigs of *Dovyalis abyssinica* collected in Kenya [84].

### 4.6. Others

The other chemical classes that have been reported in the select species include naphthoquinones, chalcones, coumarins, and glycosides, among others. Isolated chemical constituents from the selected species are shown in Table 2.

**Table 2:** Isolated chemical constituents from selected antidiabetic medicinal plants of Kenya.

Plant Species and Family	Countries in Which Samples Were Collected for Phytoconstituent Isolation	Plant Parts Used for Extraction	Isolated Chemical Compounds
*Acacia nilotica* (L.) Willd. ex Delile (Mimosoideae)	India	Stem bark	Kaempferol [37], methyl gallate [85]; catechin, gallocatechin 5-*O*-gallate, catechin 5-*O*-gallate, gallic acid, 1-*O*-galloyl-β-D-glucose, digallic acid, 1,6-di*-O*-galloyl-β-D-glucose [86]; elagic acid, (-)-Epigallocatechin-7-gallate, and (-)-epigallocatechin-5,7-digallate [87]; catechin, catechin-7-*O*-gallate, quercetin, quercetin-3-O-β- glucopyranoside, naringenin, naringenin-7-O-β-gluco-pyranoside, chalconaringenin-4′-*O*-β-glucopyronoside [86]; niloticane [88]; acanilols A and B, lupenone [89,90].
*Acalypha wilkesiana* Müll.Arg. (Euphorbiaceae)	Nigeria	Leaves, stems, root bark	Rutin, gallic acid [34,91]; corilagin, graraniin, kaempferol 3-*O*-rutinoside [92].
*Allium cepa* L. (Amaryllidaceae)	India, Egypt	Whole plant	*S*-Methylcysteine sulfoxide [93]; alliuocide G, 2-(3,4-Dihydroxybenzoyl)-2,4,6-trihydroxy-3 (2*H*)-benzofuranone, luteolin-7-*O*-D-glucopyranoside, quercetin, [1,3,11*α*-Trihydroxy-9-(3,5,7-trihydroxy-4*H*-1-benzopyran-7-on-2-yl)-5*α*-(3,4-dihydroxy-phenyl)-5,6,11-hexahydro-5,6,11-trioxanaphthacene-12-one]-4′-*O*-D-gluco-pyranoside, 1,3,11*α*-Trihydroxy-9-(3,5,7-trihydroxy-4*H*-1-benzopyran-7-on-2-yl)-5*α*-(3,4-dihydroxy-phenyl)-5,6,11-hexahydro-5,6,11-trioxanaphthacene-12-one [94]; peonidin 3′-glucoside, petunidin 3′-glucoside acetate, petunidin 3′-glucoside acetate, quercetin 3,4-diglucoside, cyanidin 3,40-di-*O*-β-glucopyranoside, isorhamnetin 3,40 diglucoside, quercetin 7-glucoside, cyanidin 40-*O*- beta-glucoside, malvidin 3′-glucoside, quercetin-3- monoglucoside, isoalliin, methiin, alliin, N-(gamma-glutamyl)-*S*-methyl-L-cysteine [95].
*Aloe secundiflora* Engl. (Asphodelaceae)	Kenya	Roots	5-Hydroxy-3,6-dimethoxy-2-methylnaphthalene-1,4-dione, laccaic acid D, 3-methoxy-2-methylnaphthalene-1,4-dione, aloesaponols I and II, chrysophanol, ancistroquinone C, helminthosporin, aloesaponarins I and II, soxanthorin, asphodelin [96].
*Carissa edulis* (Forssk.) Vahl (Apocynaceae)	Ghana, Nigeria, Kenya	Roots, fruits, leaves	Hydroxyacetophenone, catalponol, carisson, vanillin, coniferaldehyde, (-)-Nertrachelogenin, scopoletin, isofraxidin, (+)-Lariciresinol, carissanol, carinol [97]; lupeol, oleuropein, carissol [98]; 3-*O*-acetyl chlorogenic acid, kaempferol 3-*O*-β-D-glucopyranoside, quercetin-3-*O*-β-D glucopyranoside, rhamnetin-3-*O*-β-D glucopyranoside, isorhamnetin-3-*O*-β-D-glucopyranoside, (+) butyl-*O*-a-L-rhamnoside [99]; peonidin-3-rutinoside, malvidin-3-*O*-β-D-(6″-acetylglucoside) [100]; carissaedulosides A-J, [(1*S*,2*S*,3*S*)-1,2,3,4-tetrahydro-3,7-dihydroxy-1-(4-hydroxy-3-methoxyphenyl)-3-(hydroxymethyl)-6-methoxy-2-naphthalen-yl] methyl *β*-D-glucopyranoside, sarhamnoloside, (-)-lyoniresinol 9-*O*-*β*-D-glucopyranoside, khaephuoside A, (-)-lyoniresinol 9′-*O*-D-glucopyranoside, scopoletin, guaiacylglycerol, (+)-1-acetoxypinoresinol 4′-*β*-D-glucoside, acetoxypinoresinol-4′-*β*-D-glucoside 4″-*O*-methyl ether, 1-(1-hydroxyethyl)-2-(6-(1-hydroxyethyl)phenoxy)benzene, markhamioside F, 3,4-dimethoxyphenyl-2-*O*-*β*-D-apiofuranosyl-(1→2)-*β*-D-glucopyranoside [81].
*Dovyalis abyssinica* (A.Rich.) Warb. (Salicaceae)	Kenya	Leaves, twigs, roots	Dovyalicins A, B and E, *N*-(4-benzoylaminobutyl)-*N*-(3-dimethylaminopropyl)-3-phenylpropenamide, methyl 1-hydroxy-6-oxocyclohex-2-enecarboxylate, 4-hydroxy-2-(1-hydroxy-6-oxocyclohex-2-enecarbonyloxymethyl)phenyl 2-*O*-benzoyl-*α*-D-glucopyranoside, *trans*-2-{3-*O*-Acetyl-4-*O*-[(*E*)-4-hydroxycinnamoyl-*α*-D-glucopyranosyloxy}cyclohexanol [84]; benzoic acid, tremulacin, betulinic acid [101].
*Dracaena steudneri* Schweinf. Ex. Engl. (Asparagaceae)	Kenya	Leaves, fruits	Dihydrooroxylin A, 7-hydroxy-6-methoxyflavanone, 4′,5,7-trihydroxy-6-methylflavanone, 4′-*O*-methylquercetin, 3,3′-di-*O*-methylquercetin, kaempferol-3-methyl ether, jaceidin, 7-hydroxy-6-methoxyflavone, 6,8-dimethylchrysin, strobochrysin, 3,5,7-trihydroxy-6-methylflavanone, 3,5,7-trihydroxy-6-methoxyflavanone, 3,7-dihydroxy-6-methoxyflavanone, 3,5,7-trihydroxy-6-methyl-3′,4′-methylenedioxyflavone, 5,7-dihydroxy-3-methoxy-6-methyl-3′,4′-methylenedioxyflavone, 3,5,7-trihydroxy-6-methoxy-3′,4′-methylenedioxyflavone, (2S,3S)-3,7-dihydroxy-6-methoxy-3′,4′-methylenedioxyflavanone, 4′,5,7-trihydroxy-3,3′,8-trimethoxy-6-methylflavone, (2R) 7-hydroxy-2′,8-dimethoxyflavanone [45]; isorhamnetin 3-*O*-rungioside, kaempferol 3-*O*-rungioside, quercetin-3-*O*-β-D-glucoside, isorhamnetin 3-O-β-D-glucopyranoside, 3,3′ -Di-*O*-methylquercetin 4′-*O*-β-D-glucoside, quercetin, 3,3′ -Di-*O*-methyl quercetin, 4-(2ʹ-Formyl-1ʹ-pyrrolyl)butanoic acid [73]. 20,40-dihydroxy-2,30-dimethoxychalcone, kaempferol, 8-(C)-methylquercetagetin-3,6,30-trimethyl ether, alliospiroside A, methylgalangine, 6,8-dimethylchrysin, oleanolic acid, ombuine-3-*O*-rutinoside (4′,7-dimethylquercetin-3-*O*-*α*-L-rhamnopyranosyl-(1-6)-*β*-D-glucopyranoside), *β*-sitosterol 3-*O*-glucopyranoside, betulinic acid, ishigoside, and lupeol [102].
*Euphorbia hirta* L. (Euphorbiaceae)	China, India	Leaves, stems, latex	Kaempferol, afzelin, quercitrin, and myricitrin [46]; quercetin, quercetin-rhamoside, rutin [103]; isolintetralin, virgatusin, virgatusin 16, urinaligran, phyllanthin, niranthin, 5-demothoxyniranthin, lintetralin, phyltetralin, 7-hydroxy-hinokinin, 5-methoxyursehernin, hypophyllanthin, neonirtetralin, euphorhirtins A-D, 5-methoxyvirgatusin, 7*S*-ethoxyisolintetralin, 7*R*-ethoxyisolintetralin, 7*R*-ethoxy-3-methoxyisolintetralin [80].
*Euphorbia tirucalli* L. (Euphorbiaceae)	China	Leaves, stem bark, whole plant, roots	Gallic acid, dihydroxybenzoic acid, 4-*O*-methylgallic acid, ampelopsin, isoquercetin, rutin, ellagic acid, myricetin, avicularin, quercitrin, tricetin, tricetin, 3,3′-dimethoxy-4-*O*-α-rhamnopyranoside-ellagic acid, quercetin [72]; tirucadalenone, euphorol L, M, N, euphorol D, euphol, lupanone, ergosterol peroxide, vomifoliol, scopoletin, aloe-emodin [104]; 3-*O*-(2,4,68-Tetradecatetraenoyl) ingenol, 13-*O*-acetyl-12-*O*-(2*Z*,4*E*-Octadienoyl)-4β-deoxyphorbol, pedilstatin, 4β-Deoxy-phorbol-13-acetate, 4*α*-deoxy-phorbol-13-acetate, 12-*O*-(2*E*,4*E*,6*E*,8*E*-tetradecatetraenoyl)-13*-O*-isobutyroyl-4β-deoxyphorbol [67].
*Faurea saligna* Harv. (Proteaceae)	NA	NA	NA
*Lactuca inermis* Forssk (Asteraceae)	Poland	Roots, aerial parts	Scopolin, isofraxoside, 4-hydroxyphenylacetic acid, syringic acid, 9a-hydroxyzaluzanin C, 11b,13-dihydroderivative, ixerin F, 11b,13-dihydroglucozaluzanin C, α-xylofuranosyluracil [105].
*Manihot esculenta* Crantz (Euphorbiaceae)	NA	NA	NA
*Myrsine africana* L. (Primulaceae)	Kenya, China, Pakistan, South Africa	Leaves, stems	Nepodin, emodin, 5-methoxy-7-hydroxyphthalide, 2-hydroxychrysophanol [106]; myricetin-3-rhamnoside, Myricetin 3-(3″,4″-diacetylrhamnoside), myricetin 7-rharnnoside, gallic acid, myritin 3-xyloside, myricetin, myricetin 3-arabinoside, 3′-*O*-methylquercetin 3-glucoside, quercetin 3-galactoside, quercetin, kaempferol [107]; Myrsinone, embelin, 5-*O*-methylembelin, methylvilangin, methylanhydrovilangin [108]; taraxerone, taraxerol, myricadiol, stigmasterol 3-*O*-β-D-glucoside, a-spinasterol 3-*O*-β-D-glucoside [68]; muketanin [109]; myricetin 3- galactoside [110]; mearnsetin 3-(2″,4″-diacetylrhamnoside), quercitrin, myricitrin, mearnsitrin, myricetin-3-*O*-(4″-*O*-acetyl)-a-L-rhamnopyranoside, mearnsetin-3-*O*-(4″-*O*-acetyl)-α-L-rhamnopyranoside, (-)-epicatechin, (-)-epigallocatechin, (-)-epigallocatechin-3-*O*-gallate, 3′,5′-di-*C*-*β*-glucopyranosyl phloretin [111]; Myrsininones A and B [50]; Myrsigenin [112]; (3b,16a,20a)-3,16,28-trihydroxyolean-12-en-29-oic acid 3-{O-β-D-glucopyranosyl-(1-2)-*O*-[b-d-glucopyranosyl-(1-4)]-a-l-arabinopyranoside}, isolariciresinol 9′-β-D-xylopyranoside, isolariciresinol 9′-β-D-glucopyranoside, lyoniresinol 9′-β-D-glucopyranoside [113]; myricetin 3-*O*-(2″,4″-di-*O*-acetyl)-α-L-rhamnopyranoside, mearnsetin 3-*O*-(4″-Oacetyl)-α-L-rhamnopyranoside, mearnsitrin, myricetin 3-O-(4″-*O*-acetyl)-α-L-rhamnopyranoside, quercetin 3-*O*-(3″,4″-di-*O*-acetyl)-α-L-rhamnoside, rutin, quercetin 3-*O*-α-L-rhamnopyranoside, myricetin 3-*O*-α-L-rhamnopyranoside [114]; myrsinane [51].
*Persea americana* Mill. (Lauraceae)	Brazil, Taiwan, Nigeria	Leaves, seeds, fruits	Kaempferol 3-*O*-α-D-arabinopyranoside, quercetin 3-*O*-α-D-arabinopyranoside, afzelin, quercitrin, quercetin 3-*O*-β-glucopyranoside, quercetin [52]; 1,2*R*-diacetoxy-4*R*-hydroxy-*n*-heptadeca-16-ene, 2*R*,4*R*-Diacetoxy-1-hydroxy-*n*-heptadeca-16-ene, 1,2*R*-diacetoxy-4*R*-hydroxy-*n*-heptadeca-16-yne, 2*R*,4*R*-diacetoxy-1-hydroxy-n-heptadeca-16-yne, 1-acetoxy-2*R*,4*R*-dihydroxy-*n*-heptadec-16-ene, 4-acetoxy-1*R*,2*R*-dihydroxy-*n*-heptadec-16-ene, 1-acetoxy-2*R*,4*R*-dihydroxy-*n*-heptadec-16-yne, 1,2*R*,4*R*-trihydroxy-*n*-heptadec-16-yne, 1,4*R*-diacetoxy-2*R*-hydroxy-*n*-heptadeca-16-ene, 1,4*R*-diacetoxy-2*R*-hydroxy-*n*-heptadec-16-yne [115]; isorhamnetin, luteolin, rutin, quercetin, apigenin [116].
*Prunus africana* (Hook.f.) Kalkman (Rosaceae)	Switzerland, Ethiopia, South Africa	Stem bark, leaves	2α,3α-dihydroxyurs-12-en-28-oic acid, 2a,3fl-dihydroxyurs-12-en-28-oic acid, 2α,3β-dihydroxyolean-12-en-28-oic acid, 3β,24-dihydroxyurs- 12-en-28-oic acid, 2α,3α,23-trihydroxyurs-12-en-28-oic acid, 2α.3α,24-trihydroxyurs-12-en-28-oic acid, *24-O-trans-ferulyl-3fl*hydroxyurs-12-en-28-oic acid, *24-O-cis-feruly-*3β-hydroxy-urs-12-en-28-oic acid, *24-O-trans-ferulyl-*2a,3a-dihydroxy-urs-12-en-28-oic acid [117]; friedelin, ursolic acid, maslinic acid, 2 α-hydroxyursolic acid, epimaslinic acid [118]; β-sitosterol, p-hydroxybenzoic acid, oleanoic acid-3-benzoate, oleanoic acid-22-benzoate, benzoic acid [119]; β-sitosterol, β-amyrin, β-sitosterol-3-*O*-glucoside [120].
*Rhamnus prinoides* L’Hér. (Rhamnaceae)	Ethiopia	Leaves, stems, roots	Emodin, physcion, emodinanthrone, muszin, rhamnocitrin, rhamnazin, prinoidin, emodinbianthrone, chrysophanol, quercetin, rhamnetin [57]; glucofrangulin A, emodin glucoside B [121].
*Rhamnus staddo* A.Rich. (Rhamnaceae)	NA	NA	NA
*Rotheca myricoides* (Hochst.) Steane and Mabb. (Lamiaceae)	NA	NA	NA
*Trimeria grandifolia* (Hochst.) Warb. (Salicaceae)	NA	NA	NA
*Urtica massaica* Mildbr (Urticaceae)	NA	NA	NA
*Warburgia ugandensis* Sprague (Canellaceae)	Kenya, Uganda, Ethiopia	Leaves, stem bark	Kaempferide 3-O-bxylosyl (1-2)-b-glucoside, kaempferol 3-O-α-rhamnoside-7,4′-di-O-β-galactoside, kaempferol 3,7,4′-tri-O-β-glucoside, quercetin 3-O-[β-glucosyl (1-2)[α-rhamnosyl (1-6)]-β-glucoside-7-O-a-rhamnoside, quercetin, myricetin, kaempferol, kaempferol 3-rhamnoside, kaempferol 3-arabinoside, quercetin 3-rhamnoside, quercetin 3-glucoside, kaempferol 3-rhamnoside-4′-galactoside, kaempferol 3-rutinoside, myricetin 3-galactoside, kaempferol 3-glucoside [122]; ugandenial A, 11α-hydroxycinnamosmolide, polygodial, mukaadial, dendocarbin A, 9α-hydroxycinnamolide, dendocarbin L, dendocarbin M [69]; 7α-acetylugandensolide, bemadienolide, drimenin, polygodial, warburganal, ugandensidial, 6a-Hydroxymuzigadial, 9-deoxymuzigadial, ugandensolide, deacetoxyugandensolide, cinnamolide, 3β-acetoxycinnamolide [61]; muzigadial, muzigadiolide, cinnamolide-3b-acetate, linoleic acid [123]; polygodial, deacetylugandensolide [124]; nerolidol, warburgin, warburgiadione, pereniporin B, cinnamolide, cinnamolide-3 β-acetate, dendocarbin A, 9 α,11 α-dihydroxy, 6 β-acetyl-cinnamolide, dendocarbin L, 9 α-hydroxycinnamolide, 4(13),7-coloratadien-12,11-olide, 6 α,9 α-dihydroxy-4(13)-7- coloratadien-11,12-dial, 7 β-hydroxy-4(13)-8-coloratadien-11,12-olide, 7 α-hydroxy-8-drimen-11,12-olide, cinnamolide-3 β-ol, deacetylugandensolide, 11 α–hydroxymuzigadiolide [125]; *N-cis*-grossamide, *N-trans*-grossamide, 7-hydroxywinterin, 11*α-*hydroxycinnamosmolide, polygonal acid [126]; 2-[3-[2-O-(6-deoxy-α-L-mannopyranosyl)-*β*-D-glucopyranosyl]-4,5-dihydroxyphenyl]-5,7-dihydroxy-4H-1-benzopyran-4-one, 2-[3-[2-O-(6-deoxy-*α*-L-mannopyranosyl)-*β*-Dglucopyranosyl]-4-hydroxyphenyl]-5,7-dihydroxy-*4H*-1-benzopyran-4-one, 4-[(6′-O-*β*-D-allopyranosyl)-oxy]-hydroxy-benzoicacid cyclic dimeric inner ester, *N-trans*-caffeoyltyramine, 1-(3,4-dihydroxy-5-methoxyphenyl)-1,2-dihydroxy-7,8-dihydroxy-*N*-[(3,4-dihydroxyphenyl)ethyl]-*N*′-[(4-hydroxyphenyl)ethyl]-6-methoxynaphthalene-2,3-dicarboxamide, 1-(3,4-dihydroxy-5-methoxyphenyl)-1,2-dihydroxy-7,8-dihydroxy-*N*-[(4-hydroxyphenyl)ethyl]-*N*′-[(4-hydroxyphenyl)ethyl]-6-methoxynaphthalene-2,3-dicarboxamide, 1-(3,4-dihydroxy-5-methoxyphenyl)-1,2-dihydroxy-7,8-dihydroxy-*N*,*N*′-bis-[2-(4-hydroxyphenyl)ethyl]-6-methoxynaphthalene-2,3-dicarboxamide, 1-(3,4-dihydroxy-5-methoxyphenyl)-1,2-dihydroxy-6,7-dihydroxy-*N*,*N*′*-*bis-[2-(4-hydroxyphenyl)ethyl]-8-methoxynaphthalene-2,3-dicarboxamide [127].
*Zanthoxylum usambarense* (Engl.) (Rutaceae)	Kenya	Stems, roots	Usambanoline, (+)-tembetarine, (+)-magnoflorine, (-)-edulinine, (+)-*N*-methylplatydesmine, (-)-oblongine, (-)-usambarine, jatrorrhizine, (-)-*cis*-*N*-methylcanadine, nitidine, chelerythrine [64,128]; canthin-6-one, oxychelerythrine, norchelerythrine, pellitorine, (+)-2,6-bis(3,4-methylenedioxyphenyl)-3,7-dioxabicyclo[3.3.0]octane ((+)-sesamin, (+)-Piperitol-3,3-dimethylallyl ether [83].

Abbreviation: NA, not available.

## 5. Antidiabetic Activity of the Selected Medicinal Plants

### 5.1. In Vitro Antidiabetic Activity of Crude Extracts

Plant crude extracts have for a long time been prepared traditionally to prevent and treat various ailments in humans. The extracts are obtained with the use of various solvents such as water, ethanol, methanol, ethyl acetate, *n*-butanol, and chloroform, among others. Various plants extracts’ antidiabetic activities together with those of isolated compounds are shown in Table 3. Ethyl acetate and the ethanolic extracts of *A. wilkesiana* exhibited significant inhibitory activity against α-glucosidase. However, the stem bark and root bark ethanolic extracts exhibited higher activity compared to the others [33]. The same extracts also inhibited pancreatic α-amylase. The ethanolic root bark extract showed the most significant inhibition activity.

Inhibiting α-glucosidase and α-amylase enzymes involved in carbohydrate digestion can significantly reduce increases in post-prandial blood glucose levels and thus can be an important strategy for the management of blood glucose levels in type 2 diabetic and borderline patients. A study evaluating the α-amylase inhibition by the methanolic leaves extracted from *Euphorbia hirta* demonstrated that a concentration range of 25–100 µg/mL of the extract showed dose-dependent inhibition at a rate of 4.4–35.7% [1]. Shilpa et al. also evaluated the α-amylase inhibition of methanolic leaves extracted from *E. hirta*, confirming prior studies that had established that *E. hirta* inhibits α-amylase and exhibits an IC_50_ value of 0.748 mg/mL [2]. Freeze-dried juice from *E. tirucalli* has also been shown to inhibit α-amylase [129]. Altamimi et al. established that the *E. tirucalli* extract inhibited the digestive enzymes lipase and α-glucosidase with IC_50_ values of 39.8 ± 0.22 and 79.43 ± 0.38 ug/mL, respectively [129]. Ethanolic and acetone extracts of *M. esculenta* have demonstrated their anti-hyperglycemic abilities by significantly inhibiting both α-glucosidase and α-amylase in a dose-dependent manner. The study further associated the activity of the two extracts with their high level of flavonoid and phenolic contents [130]. Extracts of *D. abyssinica* have been reported to exhibit strong antidiabetic activities.

Blood glucose is a biochemical marker that is commonly utilized in the diagnosis and monitoring of diabetes mellitus. As a result of insulin deficiency, hyperglycemia develops. Diabetes mellitus is characterized by the excessive synthesis of endogenous glucose by hepatic and extrahepatic tissues via gluconeogenic and glycogenolytic pathways, as well as a decreased use of glucose by diverse organs. Treatment of streptozotocin-induced diabetic mice with an *E. hirta* methanolic leaves extract lowers blood glucose levels as well as improves insulin levels [131]. The antidiabetic effect of methanolic *E. hirta* extract could be a result of increasing insulin action by stimulating insulin release from residual pancreatic cells or from the bound form. Maurya et al. also established that the ethanolic leaf extract of *E. hirta* lowered the blood glucose levels examined of a streptozotocin-induced diabetic rat administered with a dose of 400 mg/kg body weight. Further, total cholesterol levels, and both the low- and very low-density lipoprotein levels, reduced significantly when diabetic rats were administered with the leaves’ ethanolic extract, while the high-density lipoprotein levels improved [132]. A reduced insulin production and impaired insulin action resulted in an increased lipid metabolism from adipose tissue to plasma. Diabetes mellitus is associated with an increase in cardiovascular risk due to the impaired insulin sensitivity caused by excessive lipid concentrations in the cells [132]. As a result, the altered lipid and lipoprotein patterns observed in diabetic rats could be due to insulin secretion and/or action deficiency. *A. cepa* aqueous extracts in a dose-dependent manner were established to reduce blood glucose levels and total serum lipid, as well as serum cholesterol, in alloxan-induced diabetic rats. The most effective reduction percentage for the three mentioned parameters was achieved with a dosage concentration of 300 mg/kg. The study therefore establishes that *A. cepa* exhibits notable hypoglycemic and hypolipidemic activity [133].

Lima et al. established that phospho-PKB expression in the soleus muscle significantly increased in diabetic rats treated with a *P. americana* ethanolic leaf extract. Membrane translocation and phosphorylation are processes involved in the pathway of PKB activation. The stimulation of glucose transportation by GLUT-4 translocation from the cytosol to the plasma membrane may be caused by the activation of this enzyme, which is associated with an increased absorption of glucose by the skeletal muscle, adipocytes, liver, and other tissues [134,135].

### 5.2. Antidiabetic Activity of Isolated Compounds

Plant extracts demonstrate biological activities against various diseases. The inhibition activity of the extracts towards diabetic targets varies with different concentrations. The activities of crude extracts are attributed to the synergistic potential of the phytochemicals present. Hence, isolating the chemical constituents present in these crude extracts and evaluating their biological activity is of great importance. The crude extracts of the antidiabetic plant species in this study have demonstrated significant antidiabetic activities. However, evaluation of the antidiabetic activity of the isolated compounds is lessexplored.

S-methylcysteine sulfoxide isolated from *Allium cepa* was evaluated for its antidiabetic activity in alloxan-induced diabetic rats at different concentrations and at different time levels. Upon inducing normal rats with alloxan, there was a reduction in body weight and an increase in blood sugar levels within two months. However, when treating the diabetic rats with S-methylcysteine sulfoxide, there was a significant decrease in blood sugar level and an increase in body weight within two months; thus, S-methylcysteine sulfoxide ameliorates diabetes [93]. Quercetin is a major flavonoid consumed by humans as it is widely found in most human diets. Quercetin isolated from *Allium cepa* was found to decrease the effect of streptozotocin-induced diabetes on serum MDA and improve the serum TAC levels. Further, treatment with quercetin led to a decrease in the blood sugar levels of streptozotocin-induced diabetic rats.

**Figure 3:**
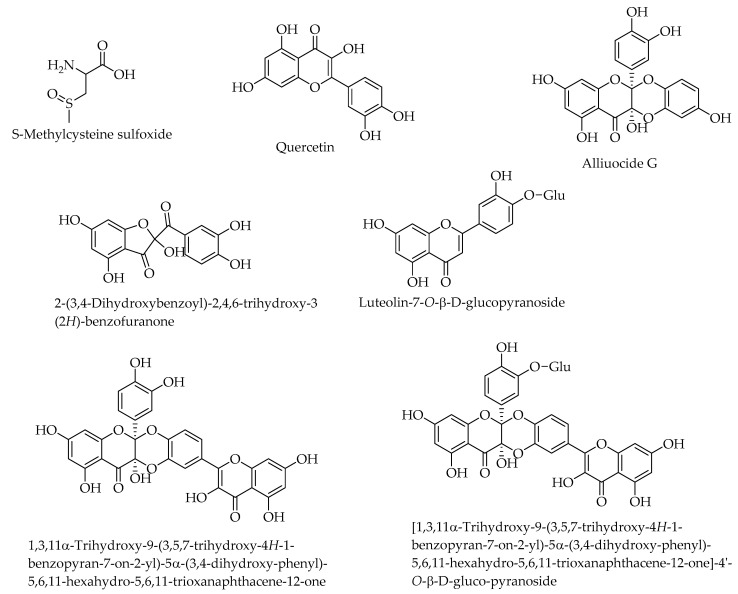
Chemical structures of antidiabetic compounds isolated from *Allium cepa*.

Alliuocide G, 2-(3,4-Dihydroxybenzoyl)-2,4,6-trihydroxy-3 (2*H*)-benzofuranone, Luteolin-7-*O*-D-glucopyranoside, quercetin, [1,3,11*α*-Trihydroxy-9-(3,5,7-trihydroxy-4*H*-1-benzopyran-7-on-2-yl)-5*α*-(3,4-dihydroxy-phenyl)-5,6,11-hexahydro-5,6,11-trioxanaphthacene-12-one]-4′-*O*-D-gluco-pyranoside, and 1,3,11*α*-Trihydroxy-9-(3,5,7-trihydroxy-4*H*-1-benzopyran-7-on-2-yl)-5*α*-(3,4-dihydroxy-phenyl)-5,6,11-hexahydro-5,6,11-trioxanaphthacene-12-one isolated from *Allium cepa* collected in Egypt demonstrated antidiabetic activity through the inhibition of α-amylase. Among them, alliuocide G exhibited the highest inhibition at a rate of 96.5% [94]. Figure 3 depicts the chemical structures of these antidiabetic chemical compounds isolated from *A. cepa*.

With it using more than 30% of overall energy expenditure and making up roughly 40% of total body mass, skeletal muscle is a significant peripheral tissue. Additionally, skeletal muscle accounts for nearly 80% of insulin-stimulated glucose transportation. Hyperglycemia can be brought on by any problems with skeletal muscle glucose absorption. As a result, insulin and skeletal muscle are crucial for preserving blood glucose homeostasis [136]. Glucose transporter GLUT4 is mostly expressed in skeletal muscle and controls the glucose absorption in both insulin-dependent and -independent pathways [137]. Therefore, the uptake of glucose depends on GLUT4 translocation to the cell membrane. Two different signaling mechanisms influence how GLUT4 moves into the cell membrane. The first is the insulin-dependent route, where insulin activates phosphatidylinositol-3 kinase (PI3K) before protein kinase B (Akt) is activated downstream and promotes GLUT4 translocation. The other is the insulin-independent adenosine 5′-monophosphate activated protein kinase (AMPK) pathway. It serves as an important cellular and systemic energy sensor and a supreme controller of metabolic balance. The activation of AMPK is triggered by increases in the AMP/ATP ratio that take place during times of energy deprivation. AMPK is an interesting and attractive therapeutic target for diseases such as diabetes and obesity. Quercetin, through activating the AMPK pathway, increases glucose uptake, plasma membrane GLUT1 protein levels, and the expression of *Glut 1* mRNA, hence demonstrating antidiabetic activity [138].

**Figure 4:**
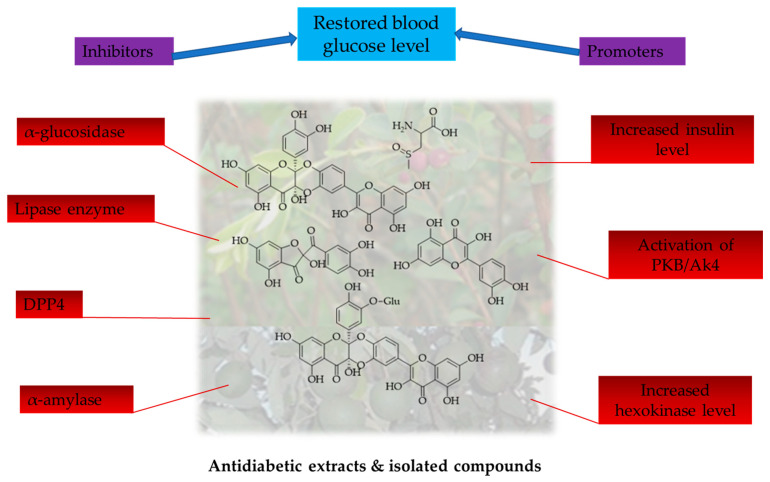
Antidiabetic mechanisms exhibited by extracts and isolated compounds from antidiabetic species.

The crude extracts of the antidiabetic species and their fractions, together with isolated chemical constituents, exhibit different antidiabetic mechanisms in the restoration of normal blood glucose levels. Figure 4 indicates these mechanisms.

**Table 3:** Crude extracts and isolated compounds from the selected medicinal species and their various antidiabetic activities.

Plant Species	Crude Extracts Tested	Isolated Compounds Tested	Antidiabetic Activities
*Acacia nilotica*	Ethyl acetate, *n-*butanol and aqueous extracts of the bark, methanol extract of pods and leaves.	NT	Hypoglycemic and antihyperglycemic effects [139].
*Acalypha wilkesiana*	Ethyl acetate and ethanol extracts of leaves, stem and root barks.	NT	Inhibition of pancreatic α-amylase [33].
*Allium cepa* L.	Aqueous and dichloromethane extracts of whole plant.	S-Methylcysteine sulfoxide, quercetin, alliuocide G, 2-(3,4-Dihydroxybenzoyl)-2,4,6-trihydroxy-3 (2*H*)-benzofuranone, luteolin-7-*O*-D-glucopyranoside,, [1,3,11*α*-trihydroxy-9-(3,5,7-trihydroxy-4*H*-1-benzopyran-7-on-2-yl)-5*α*-(3,4-dihydroxy-phenyl)-5,6,11-hexahydro-5,6,11-trioxanaphthacene-12-one]-4′-*O*-D-gluco-pyranoside, 1,3,11*α*-trihydroxy-9-(3,5,7-trihydroxy-4*H*-1-benzopyran-7-on-2-yl)-5*α*-(3,4-dihydroxy-phenyl)-5,6,11-hexahydro-5,6,11-trioxanaphthacene-12-one.	Antihyperglycemic [140], hypoglycemic, and hypolipidemic effects [133,141]. Inhbited clinical hypoglycemic activity in type 1 and type 2 diabetic patients [142]; Quercetin increased insulin levels and reduced blood sugar levels in streptozotocin-induced diabetic rats [141,143]. Alliuocide G, 2-(3,4-dihydroxybenzoyl)-2,4,6-trihydroxy-3 (2*H*)-benzofuranone, luteolin-7-*O*-D-glucopyranoside, quercetin, 1,3,11*α*-Trihydroxy-9-(3,5,7-trihydroxy-4*H*-1-benzopyran-7-on-2-yl)-5*α*-(3,4-dihydroxy-phenyl)-5,6,11-hexahydro-5,6,11-trioxanaphthacene-12-one-4′-*O*-D-gluco-pyranoside inhibition of α-amylase [94]. *Allium cepa* extracts inhibited α-glucosidase [144,145].
*Aloe secundiflora*	Aqueous extract of stem bark.		Aqueous stem bark extracts exhibited in vivo anti-hyperglycemic activity [146].
*Carissa edulis*	Ethanolic extract of leaves and methanolic extract of fruits.		Leaf extract exhibited hypoglycemic activity in streptozotocin-induced diabetic rats [147]. α-glucosidase inhibition by the fruit extracts [100].
*Dovyalis abyssinica*	NT	NT	NT
*Dracaena steudneri*	NT	NT	NT
*Euphorbia hirta*	Methanolic extract of whole plant.	NT	Methanolic extract inhibited α-glucosidase [2].
*Euphorbia tirucalli*	Aqueous stem extracts.	NT	α-glucosidase and lipase enzymes inhibitory activity [129].
*Faurea saligna*	NT	NT	NT
*Lactuca inermis*	NT	NT	NT
*Manihot esculenta*	Ethanol and acetone leaves extracts.	NT	Inhibition of α-glucosidase and α-amylase [49,130].
*Myrsine africana* L.	Methanolic leaves extract.	NT	Leaf extract reduced the levels of blood sugar in diabetes-induced albino rats [148], decreased levels of blood glucose, total cholesterol, glucose-6-phosphatase, glycated hemoglobin, fructose-1-6-bisphosphatase, and triglyceride, and increased levels of HDL cholesterol, insulin, and hexokinase [148].
*Persea americana* Mill.	Ethanolic and aqueous extracts of leaves, seeds, fruits.	NT	Leafs extract lowered blood sugar levels and improved the metabolism of diabetic rats through the regulation of glucose uptake in the liver and muscles by activating PKB/Akt and reestablishing the equilibrium of intracellular energy [134,135]. Aqueous extracts of leaves and seeds exhibited hypoglycemic effects [53,149,150]. Inhibition of α-amylase and α-glucosidase, hence lowering of post-prandial hyperglycemia [151].
*Prunus africana*	Aqueous and ethanolic stem bark extracts.	NT	Hypoglycemic effect against diabetic rats [152]. The extracts reduced the dipeptidyl peptidase-4 (DPP-4) enzyme which activates glucagon-like peptides (GLP-1) leading to insulin production in the body, hence controlling body glucose levels [153].
*Rhamnus prinoides*	NT	NT	NT
*Rhamnus staddo* (Rhamnaceae)	Aqueous extracts of leaves.	NT	Aqueous extract exhibited a hypolipidemic effect [58].
*Rotheca myricoides*	Aqueous extract of whole plant.	NT	Extracts of *R. myricoides* exhibited antihyperglycemic and antidyslipidemic effects in diabetic rats [59].
*Trimeria grandifolia*	NT	NT	NT
*Urtica massaica*	NT	NT	NT
*Warburgia ugandensis*	NT	NT	NT
*Zanthoxylum usambarense*	NT	NT	NT

Abbreviation: NT: crude extract or isolated compounds not tested for antidiabetic activities.

### 5.3. In Silico Antidiabetic Activity of Compounds

Several proteins involved in glucose metabolism have been linked to type 2 diabetes, including, Glutamine fructose-6-phosphate amidotransferase (GFPT or GFAT), Mono-ADP-ribosyltransferase sirtuin-6 (SIRT6), 11-β hydroxysteroid dehydrogenase type 1 (11β-HSD1) and protein phosphatase (PPM1B) [154]. An in silico antidiabetic study of bioactive chemical constituents (ligands) in *E. hirta Linn* targeted the mentioned proteins as receptors. The study evaluated the docking simulation of 27 chemical constituents and determined that the terpenes and flavonoids exhibited a high binding affinity to the four targeted receptors. LigandScout showed that the binding results consisted of numerous hydrogen bonds and hydrophobic interactions. Notably, due to the relatively strong hydrogen bonds of flavonoids compared to those of terpenes, they exhibited a better binding affinity to the target receptors. Compounds, myricitrin, quercitrin, taraxerol, β-amyrine, α-amyrine, pelargonium-3,5-diglucose and cyanidin-3,5-*O*-diglucose exhibited a high binding affinity among the 27 screened compounds. The study further revealed that receptor 11β-HSD1 is the best for the bioactive compounds extracted from *E. hirta* [154]. Elagic acid, quercetin, and kaempferol from *A. nilotica* were revealed in silico to contribute greatly to the plant extract’s antidiabetic activity. The study further established them to be non-toxic and non-carcinogenic [155].

### 5.4. Toxicology Study of Crude Extracts

The application of medicinal plants dates back to many decades ago and a large number of people depend on herbal medicines even to date. Evaluating the toxic implications and their safety, however, has not been prioritized yet and is crucial for human health. When aqueous crude extracts of *E. hirta* were orally administered at a dose of 400 mg/kg to 38-week mature male mice, it was observed that the ethanolic extract resulted in various degrees of testicular degeneration and a decrease in the mean seminiferous tubular diameter [103,132]. Body weight loss, changes in activity (hypo and hyperactivity), piloerection, stereotypy, diarrhea, and maternal fatalities are the most common clinical indicators of maternal poisoning [156]. In addition to morphological evaluations of viscera and organ weights during autopsies, hematological studies are commonly used to detect maternal toxicity. When the test animals were fed with *Euphorbia tirucalli* latex, the aforementioned signs were not observed nor were body weight loss or reduced organ size noticed, indicating that *E. tirucalli* does not present overt maternal toxicity [157].

### 5.5. Clinical Studies

Type 1 and type 2 diabetic patients who ingested crude extracts of *A. cepa* at a concentration of 100 g were reported to experience a reduction in their fasting blood glucose levels. The extract was also shown to reduce induced hyperglycemia in type 1 and type 2 diabetic patients [142]. The study therefore established *A. cepa* as a potential dietary supplement in the management of both type 1 and 2 diabetes mellitus.

## 6. Conclusions and Future Perspectives

Oral hypoglycemics like thiazolidines and sulfonylureas are essential components of effective diabetic treatment, however, they have adverse effects, including hypoglycemia and atherogenesis. Consequently, attempts were undertaken to find alternative therapies, including herbal medications, with minimal costs and negative effects. This has prompted studies investigating the effectiveness of herbal extracts for treating diabetes. Studies have identified several plant extracts and phytoconstituents that have hypoglycemic effects in both animal models and people. In this review that focused on twenty-two commonly used antidiabetic medicinal plant species in Kenya, their phytochemical profiles were computed as well as the antidiabetic activity for both the crude extracts and isolated chemical constituents. The majority of the screened chemical constituents in the six species belong to the class of terpenoids and flavonoids, which have been reported to demonstrate novel biological activities against various diseases.

Although the studied medicinal plants have been in use for many decades to treat diabetes and other diseases in Kenya and other countries, their phytochemistry and pharmacological activities are less studied. The review has identified crude extracts of thirteen medicinal plants out of the twenty-two selected species to exhibit strong antidiabetic activities both in vitro and in vivo while the rest have not been studied. However, more antidiabetic studies are recommended on isolated chemical constituents from the select species since only phytochemicals isolated from *A. cepa* have been examined and exhibited significant activities. Phytochemical screening on the twenty-two select species has been performed and detected many chemical constituents. Therefore, this work has gathered the pharmacological antidiabetic activities of the most commonly used antidiabetic plants in Kenya and indicated their modes of actions. Hence, the study does not only gather together the commonly used medicinal plants to treat diabetes, but also supports their usage with existing pharmacological profiles. However, research focusing on the isolation of the bioactive chemical constituents in these species is highly recommended since it has been explored less. Six of the studied species have no record of isolated chemical compounds yet they exhibit strong biological activities. Even though the antidiabetic activities of most of these species have been evaluated and demonstrate strong antidiabetic activities, much of the biological work has been evaluated on species collected from other countries. Hence, phytochemical and antidiabetic studies of these species will not only validate their traditional uses, but will also lead to the discovery of new antidiabetic drugs.

We suggest using the bio-affinity ultrafiltration liquid chromatography/mass spectrometry (UF-LC/MS) approach to discover antidiabetic compounds for isolation in order to hasten the isolation of bioactive molecules with antidiabetic properties. This method identifies ligands for specific biological targets from a mixture of chemical constituents such as crude extracts. To identify antidiabetic compounds, enzymes such as α-glucosidase can be used to identify the compounds that bind to it and hence target them for isolation studies.

## Data Availability

Not applicable.

## Sample Availability

Not available.
